# Birth preparedness and pregnancy complication readiness and associated factors among pregnant women in Ethiopia: A multilevel analysis

**DOI:** 10.1371/journal.pgph.0003127

**Published:** 2024-05-15

**Authors:** Addisalem Workie Demsash, Teshome Bekana, Sisay Yitayih Kassie, Adamu Ambachew Shibabaw, Geleta Nenko Dube, Agmasie Damtew Walle, Milkias Dugassa Emanu, Abiy Tasew Dubale, Alex Ayenew Chereka, Gemeda Wakgari Kitil, Bekem Dibaba Degefa, Aselefech Seyife, Abdurahman Mohammed Ahmed, Zenebe Abebe Gebreegziabher, Sewnet Getaye Workie

**Affiliations:** 1 Health Informatics Department, Asrat Woldeyes Health Science Campus, Debre Berhan University, Debre Berhan, Ethiopia; 2 Medical Laboratory Department, College of Health Science, Mattu University, Metu, Ethiopia; 3 Health Informatics Department, College of Health Science, Mattu University, Metu, Ethiopia; 4 Nursing Department, College of Health Science, Mattu University, Metu, Ethiopia; 5 Midwifery Department, College of Health Science, Mattu University, Metu, Ethiopia; 6 Reproductive Health Department, Asrat Woldeyes Health Science Campus, Debre Berhan University, Debre Berhan, Ethiopia; 7 Epidemiology and Biostatistics Department, Asrat Woldeyes Health Science Campus, Debre Berhan University, Debre Berhan, Ethiopia; Health Policy Research Group, University of Nigeria, NIGERIA

## Abstract

Maternal and child deaths occur during pregnancy and delivery. Timely information on signs of pregnancy complications and ways to plan for normal birth is a strategy to reduce maternal and child deaths. The purpose of this study was to assess birth preparedness, and pregnancy complications readiness and identify associated factors in Ethiopia. A cross-sectional study design was used. A total of 1635 weighted samples of pregnant women were included for analysis from the 2016 Ethiopian demographic and health survey data set. Multilevel mixed-effect logistic regression was used to estimate the effects of potential variables on birth preparedness and complication readiness. STATA version 15 software was used for data processing and analysis. A variable with a p-value < 0.05 with a 95% confidence interval was considered a significant factor. Pregnant women were informed about convulsions (8.02%), fever (35.95%), abdominal pain (28.92%), leaking fluid from the vagina (28.21%), and blurred vision (17.98%**)**. Pregnant women prepared for supplies needed for birth (38.70%), transportation (20.04%), money (18.97%), people’s support for birth (5.03%), and blood donors (3.11%). Only 56% and 44.91% of pregnant women had good birth preparedness and were informed about pregnancy complications respectively. Educational status, antenatal care visits, and region were significant factors associated with birth preparedness and complication readiness. Distance to health facility and residency were significantly associated with birth and complication readiness, respectively. Birth preparedness and complication readiness among pregnant women were low in Ethiopia. Empowering women with education, installing safe roads, building accessible health facilities, and emphasizing pregnancy complications and birth preparedness plans during antenatal care visits are important interventions to enhance birth preparedness and pregnancy complication readiness.

## Introduction

Maternal and child mortality are major public health issues in different countries [1, 2]. Though the sustainable development goals aimed to reduce maternal mortality ratio and neonatal death and to end preventable death of newborns and children [3], maternal, and child health are major public health concerns across the globe [2, 4, 5]. Globally, a total of 73% of maternal deaths are directly caused by obstetric complications, and hemorrhage, hypertensive disorders sepsis, and abortion also accounted for 27.5% of maternal deaths [6]. Though under-five child mortality declined from 93.0 deaths per 1000 live births in 1990 to 37.7 in 2019, under-five child mortality is disproportionately high in low and middle-income countries [7, 8].

Additionally, in 2019, 47% of all under-5 deaths occurred in the newborn period with about 33% of newborns dying on the day of birth [9]. These child morbidity and mortality might be caused by pregnancy and birth complications. More than five hundred thousand women die due to pregnancy complications and birth annually [10]. The majority of maternal deaths occur during labor, delivery, and the postpartum period [11].

Different socioeconomic and sociocultural factors and delays in care-seeking are major contributors to child and maternal mortality. Specifically, delays in identifying pregnancy complications, deciding to seek care, identifying and reaching a health facility for adequate and suitable treatment, saving money, and finding potential blood donors are the main contributors to pregnant women’s delays in care-seeking [12].

Therefore, skilled birth assistance [13], child vaccinations and immunizations [14], changing the behavior of pregnant women, recognizing that maternal death is preventable and avoidable, training professionals, and community mobilization are effective strategies to reduce maternal and neonatal risks [15, 16]. By following the concept of “birth preparedness, and pregnancy complication readiness (BPPCR)”, encourage pregnant women to make decisions before delivery and the potential occurrence of pregnancy complications, inform them of the signs of complications to react promptly if necessary, inform them of the locations of emergency services, and encourage pregnant women to save the money needed to pay for services and to plan their transportation [17].

When pregnant women visit a health facility, they seek information about pregnancy complications, danger signs, and birth preparedness plans as part of antenatal care (ANC) service [18]. This is a comprehensive strategy that addresses delays in seeking appropriate obstetric care, promotes prompt use of skilled healthcare services, and helps pregnant women prepare for childbirth and be ready for complications, which reduces care-seeking delays and has a positive impact on birth outcomes [19].

However, pregnant women are less likely (4.8%-17%) to be prepared for birth and complication readiness in developing countries [20, 21]. In Sub-Saharan Africa, BPPCR is not well-known and practiced [22]. According to different studies conducted in Ethiopia, pregnant women’s BPPCR is low: such as 23.3%–24.9% in Jimma town [23], 17% in Sidama [24], and 22% in Adigrat [20]. A systematic review and meta-analysis study shows that only 32% of pregnant women have prepared for birth and its complications in Ethiopia [25]. Marital status, age, occupation, history of stillbirth, educational status [20], ANC follow-up, and being pregnant for the first time [24, 26] were factors associated with birth preparedness and pregnancy complications readiness among pregnant women.

Although birth preparedness and complication readiness are critical for the enhancement of maternal and child health care, maternal morbidity and mortality, fetal health, and stillbirth are common problems due to pregnancy complications [5]. Even though many studies have been conducted about pregnant women’s BPPCR and its associated factors, the problem is still a major risk factor for maternal and child healthcare. For instance, a high number of women have given birth at home [25, 27], a low number of women have been assisted by a health professional during birth [28], and a high number of pregnant women die due to prenatal risk factors such as preeclampsia [3]. Therefore, uncovering the gap by assessing the current pregnant women’s BPPCR status and identifying factors associated with BPPCR using nationally representative data is critical to clarify that the Ethiopian demography and health survey (EDHS) is a reliable data source to gather initial insights about the problem and that further research is needed to understand the effectiveness of interventions targeted to improve birth preparedness and complications readiness. Hence, a detailed study of BPPCR and related factors is required. Finally, the study would provide credible information for policymakers and implementers to design programs and interventions to improve maternal and child health care. Additionally, this study would serve as a baseline for further similar research. Therefore, this study aimed to determine pregnant women’s birth preparedness and pregnancy complication readiness and identify associated factors in Ethiopia.

## Methods

### Ethics statement

The study was based on a secondary data source that has been publicly accessible from the Measure DHS program website (https://dhsprogram.com). Therefore, ethical approval and consent from study participants were not necessary for this study.

### Study design and setting

A cross-sectional study was conducted across the nine regions of Ethiopia. Ethiopia is located in the Horn of Africa and is bordered by Eritrea to the North, Djibouti and Somalia to the East, Sudan and South Sudan to the West, and Kenya to the South. Ethiopia has nine regional states with two administrative cities. These are subdivided into different administrative zones (817 Woredas and 16253 Kebeles).

### Data source

The 2016 EDHS dataset was used from the demographic and health survey (DHS) program website (https://dhsprogram.com). The survey was conducted by the Ethiopian Public Health Institute in collaboration with the Central Statistical Agency (CSA).

### Sampling techniques and procedures

The sampling frame used for the 2016 EDHS is a frame of all census enumeration areas (EAs) conducted by the CSA. The sample for the 2016 EDHS was designed to provide estimates of key indicators for the country. Two-stage stratified cluster sampling was used. Each region was stratified into urban and rural areas. Then, the household listing operation is used for the sampling frame of household selection in the second stage. Finally, a fixed number of households per cluster was selected. Samples were selected independently and equal proportional allocation. The distribution of women in the sampled EAs was mathematically adjusted to get a representative sample for each region in the country.

### Populations

All women aged 15–49 years were the source population. Whereas all pregnant women aged 15–49 years found in the sampled household at the time of the interview were the study population. Finally, a total of 16355 weighted samples of pregnant women were included for analysis. For more details about the methodology sections, visit the 2016 EDHS report [18].

### Variables of the study and operationalization

#### The dependent variable

Birth preparedness and complication readiness.

*Birth preparedness*. Birth preparedness of pregnant women was measured by using a set of items available from the 2016 EDHS dataset: a place of birth; supplies needed for giving birth; transportation service during an emergency; saving money or funds for an emergency; people’s support during and after birth; and potential blood donors were used to measure the birth preparedness of pregnant women during their antenatal period. Accordingly, if the pregnant women respond with at least four positive responses from the respective items, then she has a good birth preparedness plan. Otherwise, she did not have a birth preparedness plan [18, 29].

*Pregnancy complication readiness*. Pregnancy complication readiness was measured by using a set of items available from the 2016 EDHS dataset: convulsions, vaginal bleeding, severe headache, fever, abdominal pain, leaking fluid from the vagina, and blurred vision. Accordingly, if the pregnant women responded with at least four positive responses from the respective items, then women had a good pregnancy complications readiness plan. Otherwise, women did not have pregnancy complications readiness plans [18, 30, 31].

#### Independent variables at the individual level

The individual-level independent variables of age, sex, educational status, respondents’ current working status, ANC visit, place of delivery, media, family size, and wealth status were used to assess birth preparedness and pregnancy complications readiness of pregnant women.

*ANC visit*. The pregnant women had visited a health facility during their pregnancy for ANC services. Accordingly, if the women had four or more visits during their pregnancy, then they had an “adequate ANC visit = 1” and if the women visited the health facility at most three times, then they had “inadequate ANC visit = 0” [32, 33].

*Media exposure*. Households that have owned either radio or television were considered that the households had media exposure. Otherwise, households had no media exposure [32].

#### Community level variables

The community-level independent variables of residence, region, and distance to health facilities were used to assess birth readiness and pregnancy complication readiness.

*Distance to a health facility*. Distance to a health facility is the main factor for accessing health care services. Therefore, healthcare services were considered to be accessible if the average walking distance to the nearby health facility was less than 2 hours [34]. Otherwise, not accessible.

#### Data management and statistical analysis

Data cleaning was performed to prepare the data for analysis according to the objectives of the study. The variables were re-coded to meet the desired classification. To ensure the representativeness of survey results at the national level [35, 36], sampling weights were calculated before analysis. STATA version 15 software was used for data management and statistical analysis.

#### Multilevel mixed effect logistic regression analysis

Since the nature of the EDHS dataset was hierarchical in nature, the records within the cluster might be dependent (the response of one respondent might be similar to another’s respondent) which disturbs the assumption of independence. A biased statistical report might be generated by fitting a model with correlated data. So, multilevel mixed effect logistic regression analysis was assumed to be used to generate and report good results. To assess dependency between the clusters, four models have been set: **model** A (a null model that assesses BPCR within the cluster), **model B** (Contains individual-level variables), **model C** (contains community-level variables), and **model D** (the aggregate model of **models B** and **C**). The intraclass correlation coefficient (ICC) was calculated to check the dependency within the cluster. If the ICC value is >0.25, the data is fitted for a multilevel mixed-effect logistic regression model [37]. Consequently, 41% and 28.5% of the ICC values (**Tables 2 and 3**) confirmed that there was significant dependency (correlations) among pregnant women within the cluster on birth preparedness and pregnancy complication readiness, respectively. Therefore, a multilevel mixed-effect logistic regression analysis model was employed to alleviate the correlations within the cluster among pregnant women on their birth preparedness and pregnancy complication readiness. A likelihood ratio (LLR) was used for model comparison, and the model with the highest value was taken as the best-fit model to solve the correlation within the cluster [38, 39]. As a result, **model D** was chosen as the best-fit model due to its LLR score’s highest value (-970.1, 983), for both dependent variables (**Tables 2, 3**). In multilevel multivariate mixed effect logistic regression analysis, a P-value less than 5% with a 95% CI was used to identify factors associated with birth preparedness and pregnancy complication readiness among pregnant women in Ethiopia.

## Results

### Sociodemographic characteristics of the study

A total of 1635 weighted samples of pregnant women were used in this study. Almost three-fourths (76.7%) of the pregnant women were rural residents. One-fourth (25.0%, 25.6%, and 25.9%) of the pregnant women were from the Amhara, Oromia, and SNNPR regional states of Ethiopia, respectively. Three out of ten (29.5%) and nearly one-fourth (24.1%) of the pregnant women were under the age groups of 25–29 and 30–34, respectively. The majority (83.9%) of the household heads were male, and half (49.5%) of the pregnant women didn’t have any formal education. Around half (51.7%) of the pregnant women were rich in wealth status (**Table 1**).

**Table 1:** Sociodemographic characteristics of the study participants, 2016 EDHS data sets.

Variable	Category	Frequency (n)	Percent (%)
Place of residency	Urban	381	23.30
	Rural	1254	76.70
Region	Tigray	219	13.40
	Afar	5	.30
	Amhara	408	25.00
	Oromia	418	25.60
	Somali	25	1.50
	Benishangul-Gumuz	19	1.20
	SNNPR	423	25.90
	Gambela	4	.20
	Harari	6	.40
	Addis Ababa	100	6.10
	Dire Dawa	6	.40
Women’s age in the year	15–19	56	3.40
	20–24	325	19.90
	25–29	482	29.50
	30–34	394	24.10
	35–39	237	14.50
	40–44	106	6.50
	45–49	35	2.10
Highest educational status	No formal education	810	49.50
	Primary	526	32.20
	Secondary	181	11.10
	Higher	118	7.20
Sex of household head	Male	1372	83.90
	Female	263	16.10
Households’ wealth status	Poor	473	29.00
	Middle	316	19.30
	Rich	845	51.70

### Pregnancy complication and birth preparedness plan by region, 2016 EDHS

**Fig 1:**
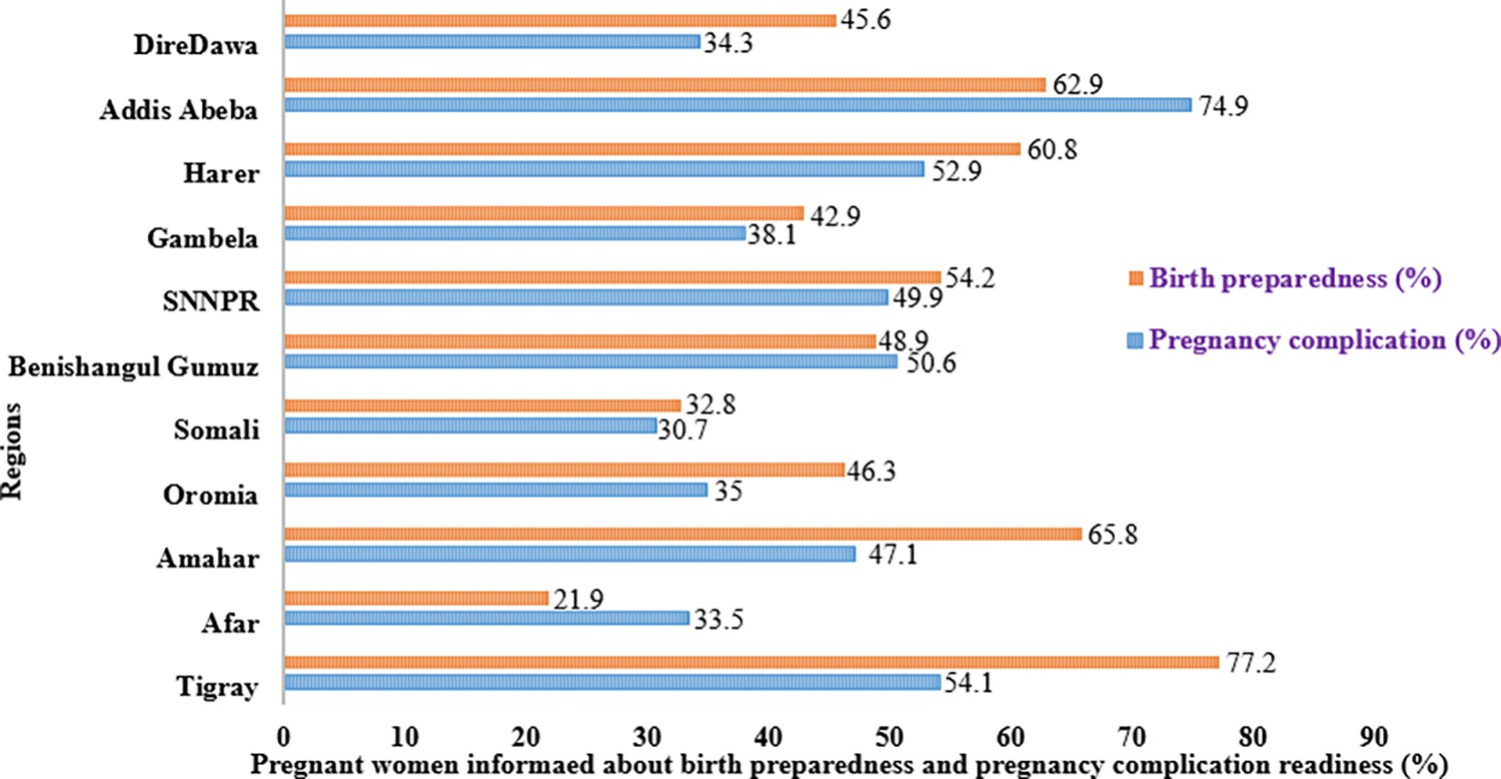
Number of pregnant women informed about pregnancy complications and birth preparedness plan during their ANC visit, 2016 EDHS.

Three-fourths (74.9%) of pregnant women who lived in the Addis Ababa region had informed about pregnancy-related complications during their ANC visits, and the majority (77.2%) of pregnant women who lived in the Tigray region had informed about birth preparedness plans. Relatively, pregnant women who lived in Afar, Somali, Gambela, Dire Dawa, and Oromia had low pregnancy complications and birth preparedness plans (**Fig 1**).

### Pregnant women informed about signs of pregnancy complications in Ethiopia

**Fig 2:**
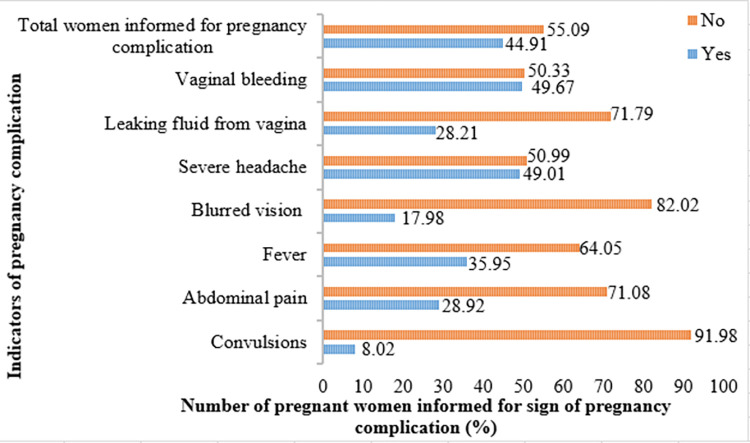
Women informed about the signs of pregnancy complications in Ethiopia, 2016 EDHS.

The 2016 EDHS data on the component of ANC was used and analyzed to determine whether the mother was informed about pregnancy complications for need of birth preparedness plan. Among the pregnant women who had live births in the 5 years before the survey, 44.91% (95% CI: 42.48%–47.30%) of pregnant women were informed of about signs of pregnancy complications during their ANC visits. Among these, 8.02% about convulsions; 49.67% about vaginal bleeding; 49.01% about a severe headache; 35.95% about fever; 28.92% about abdominal pain; 28.21% about leaking fluid from the vagina; and 17.98% about blurred vision (**Fig 2)**.

### Birth preparedness plan of women in Ethiopia

**Fig 3:**
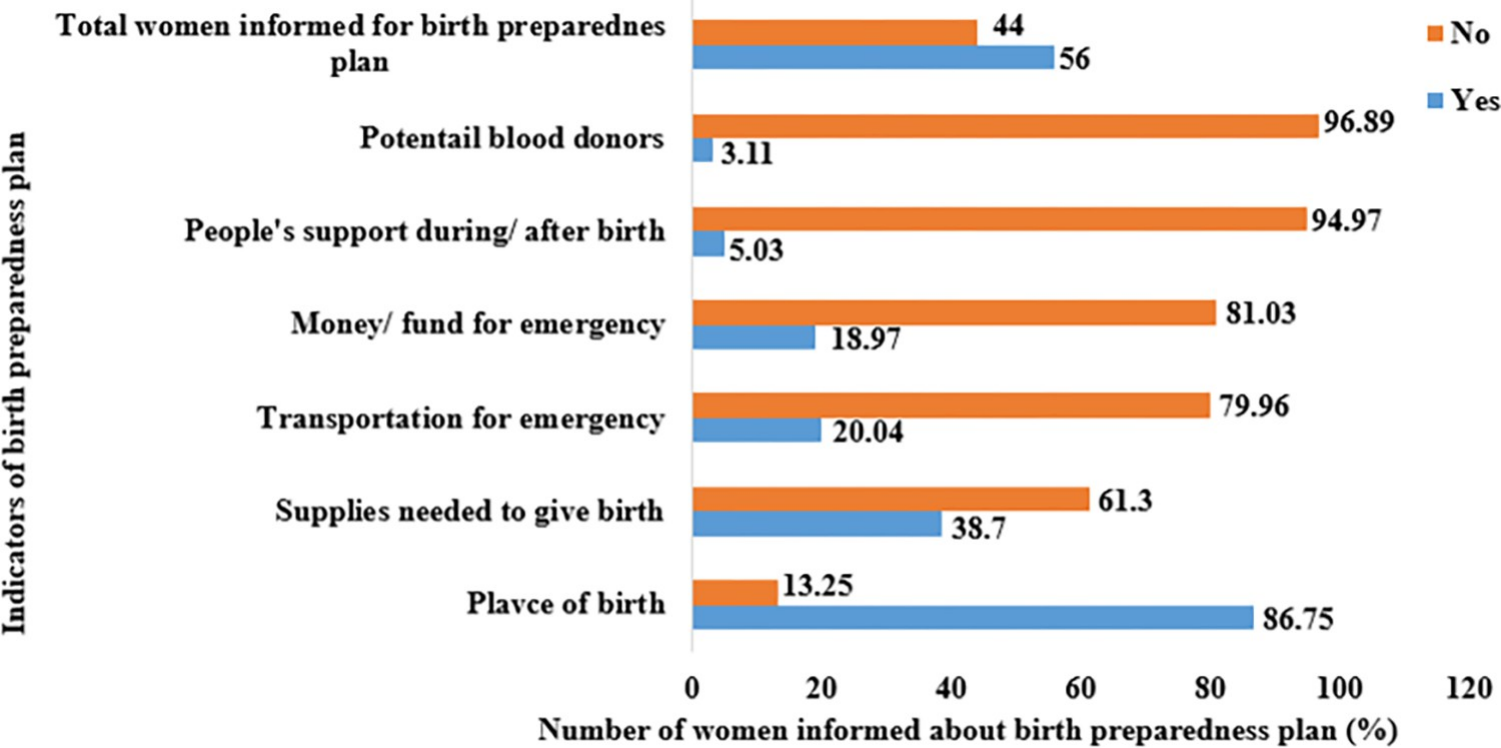
Birth preparedness plan of women in Ethiopia, 2016 EDHS.

Among pregnant women who received ANC service for their recent live birth, 56% (95% CI: 53.56%-58.37%) were informed about the birth preparedness plan. The majority (86.75%) of women were informed about the place of birth, 38.70% about supplies needed for giving birth, 20.40% about emergency transportation, 18.97% about money or funds for emergencies, 5.03% about people’s support during and after birth, and 3.11% about potential blood donors (**Fig 3**).

### Factors associated with birth preparedness among pregnant women

In the multivariate multilevel mixed effect logistic regression analysis, educational status, ANC visits, and region were statistically significantly associated with pregnant women’s birth preparedness and pregnancy complication readiness. Distance to healthcare facilities and place of residence were statistically significant predictors of pregnant women’s readiness for birth and pregnancy complications.

Women who had adequate ANC visits were 1.4 (AOR: 1.38, 95% CI: 1.13, 1.96) and 1.3 (AOR: 1.25, 95% CI: 1.38, 2.81) times more likely to be informed of birth-preparedness and pregnancy complication readiness than women who had inadequately ANC visits, respectively. Women at higher education levels were 1.8 (AOR: 1.83, 95% CI: 1.18, 2.88) and 1.7 (AOR: 1.70, 95% CI: 1.14, 2.88) times more likely to be informed about birth-preparedness and pregnancy complication readiness than women who had no formal education, respectively. Women with secondary education were also 1.3 (AOR: 1.27, 95% CI: 1.90, 2.83) times more likely to be informed about pregnancy complications than women with no formal education (**Tables 2 and 3**).

**Table 2:** Multivariable multilevel mixed effect logistic regression analysis of factors associated with birth preparedness among pregnant women in Ethiopia, 2016 EDHS data sets.

Variables	Category	Model A	Model B	Model C	Model D
			AOR (95% CI)	AOR (95% CI)	AOR (95% CI)
Media	Yes		1.13 (.84, 1.48)	-	1.12 (.83, 1.46)
	No		1		1
Educational status of women	Primary		1.18 (.89, 1.66)		1.12 (.79, 1.64)
	Secondary		1.33(.91, 1.95)		1.32 (.90, 1.95)
	Higher		1.84 (1.19, 2.92)^b^		1.83 (1.18, 2.88)^a^
	No education		1		1
Sex of household head	Female	-	0.22 (.25, .81)^b^	-	0.21 (.23, 1.03)
	Male	-	1	-	1
Currently working	Yes		1.37 (1.02, 1.85)^b^	-	1.36 (.98, 1.84)
	No		1	-	1
ANC visit	Adequate	-	1.44 (1.21, 1.98)^b^	-	1.38 (1.13, 1.96)^a^
	Inadequate	-	1	-	1
Family size	≥5	-	1.22 (.95, 1.75)^b^	-	1.21 (.85, 1.72)
	<5	-	1	-	1
Place of delivery	Institutional	-	1.09 (.83, 1.44)^b^	-	1.08 (.81, 1.43)
	Home	-	1	-	1
Wealth	Rich		1.06 (.83, 1.63)	-	0.97 (.83, 1.61)
	Middle		1.88 (.68, 1.95)	-	1.81 (.62, 1.91)
	Poor		1		1
Region	Afar		-	0.29 (.10, .95)^b^	0.26 (.10, 1.03)
	Amhara		-	0.23 (.09, .71)^b^	0.22 (.08, .70)^a^
	Oromia		-	.59 (.28, 1.24)	0.58 (.22, 1.18)
	Somali		-	.32 (.13, 1.41)	0.31 (.12, 1.21)
	Benishangul		-	.33 (.15, .72)^b^	0.31 (.14, .69)^a^
	SNNPR		-	1.33(.66, 2.66)	1.31 (.64, 2.63)
	Gambela		-	.71 (.29, 1.69)	0.70 (.29, 1.68)
	Harari		-	.29 (.12, .60)^b^	0.28 (.12, .59)^a^
	Addis Ababa		-	1.42 (.62, 3.23)	1.39 (.55, 3.21)
	Dire Dawa		-	1.75 (.65, 4.77)	1.73 (.64, 4.75)
	Tigray			1	1
Residency	Urban	-	-	0.66 (0.41, 1.07)	0.65 (.40, 1.04)
	Rural			1	1
Distance to a health facility	No problem			1.62 (1.19,2.22)^b^	1.58 (1.18, 2.21)^a^
	Problem			1	1
Variance		0.41	0.34	0.38	0.39
LLR		-1022.5	-1019.7	-991.8	-970.1
ICC		0.32	0.329	0.383	0.385

b = significant at model B and C, a = significant at model D

**Table 3:** Multivariable multilevel mixed effect logistic regression analysis of factors associated with pregnancy complication readiness among pregnant women in Ethiopia, 2016 EDHS data sets.

Variables	Category	Model A	Model B	Model C	Model D
			AOR (95% CI)	AOR (95% CI)	AOR (95% CI)
Media	Yes		1.13 (.84, 1.48)	-	1.12 (.84, 1.46)
	No		1		1
Educational status of women	Primary		1.18 (.89, 1.66)		1.15 (.86, 1.61)
	Secondary		1.33(1.91, 2.95)^b^		1.27 (1.90, 2.83)^a^
	Higher		1.71 (1.19, 2.92)^b^		1.70 (1.14, 2.88)^a^
	No education		1		1
Sex of household head	Female	-	0.97 (.75, 1.31)^b^	-	1.09 (.73, 1.31)
	Male	-	1	-	1
Currently working	Yes		1.37 (1.06, 2.53)^b^	-	1.36 (.98, 2.49)
	No		1	-	1
ANC visit	Adequate	-	1.44 (1.38, 2.98)^b^	-	1.25 (1.37, 2.81)^a^
	Inadequate	-	1	-	1
Family size	≥5	-	1.22 (1.02, 2.75)^b^	-	1.21 (.94, 2.73)
	<5	-	1	-	1
Place of delivery	Institutional	-	1.19 (1.03, 2.44)^b^	-	1.06 (.98, 2.43)
	Home	-	1	-	1
Wealth	Rich		1.16 (.83, 1.63)	-	1.17 (.63, 1.54)
	Middle		1.88 (.68, 1.95)	-	1.81 (.62, 1.71)
	Poor		1		1
Region	Afar		-	0.29 (.10, .95)^b^	0.27 (.12, 1.05)
	Amhara		-	0.23 (.09, .97)^b^	.22 (.08, 1.02)
	Oromia		-	.59 (.28, 1.24)	.54 (.21, 1.23)
	Somali		-	.32 (.13, .81)	2.54 (1.36, 4.76)
	Benishangul		-	.33 (.15, 1.72)	0.28 (0.24, 1.48)
	SNNPR		-	1.33(.66, 2.66)	1.27 (1.32, 2.64)
	Gambela		-	.71 (.29, 1.69)	.82(0.20, 1.62)
	Harari		-	.29 (.18, .60)^b^	.28 (.15, 1.05)
	Addis Ababa		-	2.72 (1.62, 3.83)^b^	2.71 (1.61, 3.81)^a^
	Dire Dawa		-	1.75 (.65, 4.77)	1.73 (.63, 4.75)
	Tigray			1	1
Residency	Urban	-	-	1.56 (1.41, 2.87)^b^	1.35 (1.44, 2.58)^a^
	Rural			1	1
Distance to a health facility	No problem			1.62 (0.96,2.22)	1.58 (.95, 2.21)
	Problem			1	1
Variance		0.39	0.31	0.32	0.33
LLR		-1505	-1041	-989	-983
ICC		0.285	0.244	0.256	0.273

b = significant at model B and C, a = significant at model D

The women who walked for less than two hours on average to the nearby health facilities were 1.6 (AOR: 1.58, 95% CI: 1.18, 2.21) times more likely to be informed about birth preparedness than women who walked more than two hours on average. Women in the Ethiopian regions of Amhara, Benishangul, and Harari were 78% (AOR: 0.22, 95% CI: .08, .07), 69% (AOR: 0.31, 95% CI: .14, .69), and 72% (AOR: 0.28, 95% CI: .12, .59) less likely to be informed about birth preparedness, respectively (**Table 2**).

Urban women were 1.4 (AOR: 1.35, 95% CI: 1.44, 2.58) times more likely to be informed about pregnancy complications than rural women. The women who lived in Addis Ababa were 2.7 (AOR: 2.71, 95% CI: 1.61, 3.81) times more likely to be informed about pregnancy complications readiness plans (**Table 3**).

## Discussion

Among women who were informed of pregnancy complications during their ANC visits, a small number of pregnant women were informed of convulsions (8.02%), fever (35.95%), abdominal pain (28.92%), leaking fluid from the vagina (28.21%), and blurred vision (17.98%). These findings were in line with studies done in Nigeria [40], and Tanzania [31]. However, around half of the pregnant women were informed about vaginal bleeding (49.67%) and severe headache (49.01%). Pregnant women were also informed about their recent birth preparedness plans, such as place of birth, supplies needed during birth, saving money for transport and emergency, blood donors, and people’s support during and after birth. These are all critical steps for pregnant women to find a skilled provider and seek care. Therefore, 86.50% of pregnant women identified their place of birth in this study. This evidence was similar to the studies done in Northern Ethiopia (75%) [20], and Tanzania [31]. Pregnant women were also identified on other birth preparedness plan items such as supplies needed for giving birth (38.70%), emergency transportation (20.04%), money or funds for emergencies (18.97%), people support during and after birth (5.03%), and potential blood donors (3.11%). Overall, 56%, and 44.91% of pregnant women were informed about their birth preparedness, and pregnancy complications during their ANC visits. These findings were higher than studies done in India (47.80%) [26], and Uganda (35%) [16]. However, the findings about birth preparedness were lower than the study done in Rwanda [41]. This might be due to low-quality health service provision at government health facilities [26], women might not have good awareness of safe delivery, and they might have low understanding of counseling messages during ANC visits [20]. The content of the counseling message in routine practices might not meet the needs of the users in local settings [42]. Additionally, the low educational status of women in this study, women’s and men’s participation in group discussions about BPPCR [43], and the absence of funding groups in the community might be possible reasons. Additionally, the districts might be located in areas where roads and ambulances could not be accessible and reachable through waking, and many women might plan to give birth at home as it is a common problem in low-income countries [44]. Moreover, women’s restriction on decision-making power and fear of hearing pregnancy complications might be important reasons behind the low BPPCR plan of pregnant women in Ethiopia [45].

The pregnant women who had adequate ANC visits were 1.4 and 1.3 times more likely to be informed about birth preparedness and pregnancy complication readiness than their counterparts, respectively. This finding was supported by studies done in Ethiopia [46], India [26], Tanzania [47], and Nigeria [22]. This might be due to women who had health facility visits being exposed to the relevant program, receiving some form of counseling, and getting effective intervention mechanisms to promote better birth preparedness and complication readiness [26]. Women who had health facility visits might be more likely to access health services and exposed to various health promotion programs [48]. Early and frequent visits to health facilities might identify and alleviate the risk factors in pregnancy and encourage women to prepare for birth through periodic and repeated counseling [49]. So, pregnant women who visit a health facility at least four times might have more opportunities for birth preparedness and complication readiness.

The women with higher education status were 1.8 and 1.7 times more likely to be informed about birth preparedness and pregnancy complication readiness than women who had no formal education, respectively. Women with secondary educational status were 1.3 times more likely to be informed about birth complications readiness than women who had no formal education. This finding is in line with studies done in Northern Ethiopia [20], Uganda [30], and Tanzania [31]. This might be due to educated women being more likely to decide on their health and be better aware of obstetric complications [31]. Plus, women who knew the danger signs of obstetric complications might essentially seek skilled birth attendants [50] and better understand the health messages acquired from numerous sources [30].

The women who lived in the Amhara, Benishangul, and Harari regions were 78%, 69%, and 72% less likely to be prepared to give birth, respectively. The possible reasons might be an inadequate number of ANC visits, less attention given to key danger signs while giving health education and counseling, and limited access to information in these regions [23]. Moreover, in this region health services might not be available and accessible, and women’s health information access and health care-seeking behavior might be inconsistent [51].

The women who mentioned that distance to a health facility was not a problem were 1.6 times more likely to be prepared to give birth than women who mentioned distance to a health facility was a problem. This finding is supported by studies done in Ethiopia [23], and Nigeria [40]. This might be due to the unavailability of transportation services for pregnant women. Many pregnant women might access dangerous and unsafe types of transportation (motorcycles) that lead to accidents [52]. Lack of money and inaccessibility of roads are barriers to reaching health facilities and seeking care [12]. Women might be poor, and the saved money might not be able to pay for transport [20]. For instance, only 20.04% and 18.97% of women were prepared for emergency transportation and saved money for emergencies for their birth. Therefore, pregnant women who are far from the health facility might be delayed in seeking care and less prepared to give birth.

Urban women were 1.4 times more likely than rural women to be informed about pregnancy complications. This finding is the same as studies done in Bangladesh [53], and Nigeria [40]. This might be due to the accessibility of healthcare facilities in urban areas, the uneven distribution of health services and healthcare professionals in rural areas, the limited access to health information, and women in rural areas might face transportation problems to reach healthcare facilities [12, 20]. Additionally, urban women are more aware of the dangerous signs of pregnancy, labor, and the postpartum period, and unpreparedness for birth among rural pregnant women is common and might invite more maternal and neonatal deaths [53]. Surprisingly, the women who lived in Addis Ababa were also 2.7 times more likely to be ready for pregnancy-related complications. As we reason out why urban women are more likely to be informed about pregnancy-related complications, this is because pregnant women who reside in urban areas like Addis Ababa have adequate prenatal care coverage [54].

### Strengths and limitations of the study

The evidence reported in this study is representative of the study subjects since it was done using nationally representative data. Conducting a multilevel mixed-effect logistic regression model alleviates the effects of the cluster on the dependency of observations. Since the data was collected retrospectively, it may be prone to recall bias.

## Conclusions and recommendations

A limited number of women were informed about pregnancy convulsions, fever, abdominal pain, leaking fluid from the vagina, and blurred vision. Around half of the pregnant women were informed of vaginal bleeding and severe headaches. A small number of pregnant women were prepared for supplies needed for giving birth, emergency transportation, money or funds for emergencies, people’s support during and after birth, and potential blood donors to give birth.

Educational status, ANC visits, and region were statistically significant factors associated with birth preparedness and pregnancy complication readiness. Distance to health facilities and place of residency were statistically associated with birth preparedness and pregnancy complication readiness, respectively.

Empowering women with education, and emphasis on BPPCR plans during women’s visits to the health facility, building nearby health facilities, safe and accessible road installation, arrangement of transport services such as ambulance, and fair and equal health services distribution across the regions are recommended interventions for maternal and child health care. Additionally, high-quality research on BPPCR would be required.
